# Associations and Potential Pathways Linking Excessive Short-Video Use to Mental Health Among Vocational High School Students in China: An Integrated Analysis Based on Machine Learning and Path Analysis

**DOI:** 10.3390/bs16081429

**Published:** 2026-08-19

**Authors:** Qihan Zhang, Baidong Huang, Hongwen Song

**Affiliations:** 1 Faculty of Psychology, Tianjin Normal University, Tianjin 300387, China; zhangqihan@tjnu.edu.cn (Q.Z.); huangbaidong@stu.tjnu.edu.cn (B.H.); 2 Key Research Base of Humanities and Social Sciences of the Ministry of Education, Academy of Psychology and Behavior, Tianjin Normal University, Tianjin 300387, China; 3 Faculty of Life Sciences and Medicine, University of Science and Technology of China, Hefei 230026, China

**Keywords:** excessive short-video use, mental health, vocational high school students, social support, coping styles

## Abstract

Excessive short-video consumption is a global concern, yet its association with mental health among vocational high school students in China remains under-researched. Among many candidate individual, psychological, and behavioral factors, it remains unclear which are the most robust predictors of mental health. This study develops a comprehensive framework to identify core predictors and examine potential pathways linking short-video use to mental health. A survey was administered to 8346 vocational high school students recruited from vocational high schools in Tianjin, China, yielding 6096 valid responses for multi-stage analysis. First, Random Forest (RF) and linear Support Vector Regression (SVR) screened 13 individual, psychological, and behavioral features to identify core predictors. Subsequently, a Path Analysis (PA) with the Bootstrap method was used to examine statistical mediating pathways involving these core features. Across RF and SVR, social support, excessive short-video use, positive coping, and negative coping emerged as the four most important predictors of mental health. PA showed generally good fit (χ^2^/df = 5.86, CFI = 0.998, TLI = 0.99, RMSEA = 0.03, SRMR = 0.01). Excessive short-video use was significantly and directly associated with mental health problems (effect = 0.221, 95% CI = [0.189, 0.253], 76.7% of the total association) and showed small indirect associations through three statistical pathways: (1) lower social support (effect = 0.023, 8.1%); (2) higher negative coping (effect = 0.035, 12.0%); and (3) a small serial indirect association from reduced social support to decreased positive coping (effect = 0.007, 2.6%). Findings suggest that potential intervention efforts may consider regulating short-video use while simultaneously enhancing social support and adaptive coping mechanisms as part of strategies to reduce psychological risks.

## 1. Introduction

With the in-depth penetration of digital technologies, the Internet and online social platforms have emerged as the core infrastructure underpinning contemporary life. Nevertheless, their associated negative ramifications are becoming increasingly apparent. The World Health Organization (WHO), health authorities across multiple nations, and meta-analytic studies have all explicitly established that excessive social media usage is closely correlated with a rise in mental health issues among adolescents, thereby constituting a public health challenge that demands urgent attention (Shannon et al., 2022; World Health Organization, 2020, 2024). Notably, this concern is particularly relevant to short-form videos. Short-video platforms have become a widely adopted and highly engaging form of social media. In addition to retaining the social features of traditional social media, these platforms employ full-screen immersive interfaces, algorithmically personalized recommendations, and “infinite scroll” designs, which may encourage prolonged and repetitive use (China Internet Network Information Center, 2024; Tian et al., 2023). Such features may facilitate “flow” experiences and problematic dependence in users and have been associated with adolescents’ cognitive functioning and mental well-being (Nguyen et al., 2025).

### 1.1. Vocational High School Students in the Chinese Educational Context

In China, after completing nine years of compulsory education, students take the senior high school entrance examination and subsequently enroll in either general senior high schools or vocational high schools. Vocational high schools generally enroll students aged 15–18 and consist of a three-year program that integrates general academic coursework, vocational skills training, and practical instruction. Upon graduation, students may either enter the labor market or pursue higher vocational education (OECD, 2020). According to data released by the Ministry of Education of China (2024), China had 15,381 general senior high schools enrolling approximately 28.04 million students in 2023, as well as 7085 vocational high schools enrolling approximately 12.98 million students. The latter accounted for roughly one-third of all students at the senior secondary level. Thus, vocational high school students constitute a large and important segment of the adolescent population in China.

In addition to the academic, interpersonal, and identity development pressures commonly experienced by adolescents, vocational high school students must balance general academic coursework, vocational skills training, and practical training. They are also expected to undertake multiple developmental tasks at a relatively early age, including selecting a field of specialization, developing a vocational identity (i.e., a clear, committed sense of oneself in relation to a future occupation), and planning for further education and employment (Cui et al., 2026; OECD, 2020). At the same time, vocational education continues to occupy a stigmatized position within China’s educational tracking system, as reflected in the negative labeling, social distancing, denigration, and discrimination experienced by its students (Wang et al., 2022). Such negative evaluations may also be internalized as self-stigma, undermining students’ self-evaluations, sense of belonging, and expectations for the future (Leng et al., 2025). Taken together, these circumstances expose vocational high school students to heightened levels of stress. Compensatory Internet Use Theory proposes that when individuals experience stress, negative emotions, social exclusion, or unmet psychological needs in their offline lives, they may turn to online activities for emotion regulation, temporary escape, and psychological compensation (Kardefelt-Winther, 2014). Through algorithmic recommendations, continuous playback, immediate feedback, and highly entertaining content, short-form video platforms can provide low-cost distraction and short-term gratification and may therefore serve as a compensatory means of coping with stress among vocational high school students. Direct research on excessive short-form video use in this population remains scarce. Nevertheless, studies of internet and smartphone addiction have reported high prevalence rates among vocational high school students and adverse associations with their mental health (Fan et al., 2025). For example, Zhai et al. (2025) surveyed 4897 vocational high school students in Shanghai and found that the prevalence of smartphone addiction was as high as 70.5%. In a study of vocational high school students in Hunan Province, Teng et al. (2023) reported lifetime prevalence rates of 37.7%, 15.7%, and 21.8% for suicidal ideation, suicide plans, and suicide attempts, respectively, and further identified a significant association between internet addiction and suicidal behaviors. Because the personalized recommendations and immediate-reward mechanisms of short-form video platforms may continually reinforce compensatory use, vocational school students facing substantial real-life stress may be particularly susceptible to progressing from short-term emotion regulation to excessive use, with attendant risks to mental health. This possibility, however, remains to be empirically tested. It should be noted that the evidence discussed above is derived primarily from Chinese vocational students, whereas the short-video platforms used, modes of engagement, and vocational education experiences may differ across cultural contexts.

### 1.2. Potential Pathways Linking Excessive Short-Video Use to Mental Health

Building on research with general adolescent and adult populations, scholars have sought to deconstruct the potential pathways through which excessive short-video (or social media) use is associated with mental health across multiple dimensions, with a primary focus on the following three pathways. First, the psychological mechanism path. Based on the I-PACE model proposed by Brand et al. (2016, 2019), excessive short-video use is regarded as a maladaptive avoidance coping strategy. It may be associated with individuals’ mental health problems through lower cognitive control resources and less adaptive emotion regulation abilities. For instance, Yao et al. (2026) found a bidirectional relationship between excessive short-video use and difficulties in emotion regulation. Y. Liu et al. (2021) discovered that individuals use short videos to escape from real-life stress and self-soothe, which is a typical avoidance coping mechanism. Coping styles and emotion regulation are core predictors of the occurrence and maintenance of mental health problems (Aldao et al., 2010; Compas et al., 2017). Additionally, individuals lacking real-life social support are more inclined to seek virtual companionship or social interaction through short videos (Zhao & Kou, 2024). However, excessive immersion in the virtual world further isolates real social interaction, which may coincide with lower real-world social support and greater psychological difficulties (Kraut et al., 1998). Thus, excessive use of short videos may be associated with individuals’ mental health through these psychological factors. Second, the individual differences path. Gender differences, socioeconomic status, and other factors have been proven to significantly moderate the strength of the association between short-video usage behavior and mental health (Jain et al., 2025; Wu et al., 2025). For example, a meta-analytic study by Jain et al. (2025) demonstrated that females and individuals with lower socioeconomic status (SES) exhibit a higher vulnerability to short-video addiction. Third, the behavioral substitution path. According to the Time Displacement Hypothesis, excessive immersion in short videos often crowds out time for physical exercise and academic engagement (Jain et al., 2025; Röhlke, 2025), and may further relate to mental health through reduced physical fitness and academic setbacks (Monzonís-Carda et al., 2024; Rodriguez-Ayllon et al., 2019).

However, the existing research still has certain limitations. Firstly, the aforementioned studies have mainly focused on general adolescent or adult populations, and whether the related pathways are observed among vocational students remains to be examined. Secondly, most of the achievements are limited to the analysis of a few variables or simple relationships, lacking an overall perspective that integrates multiple levels such as individual, psychological and behavioral aspects. Moreover, traditional approaches typically pre-specify a small set of variables, making it difficult to determine, in a data-driven manner, which factors are the most robust predictors among many candidates.

### 1.3. The Present Study

In view of this, this study constructs a comprehensive analytical framework to examine the associations and potential pathways linking excessive use of short videos with mental health. In this framework, excessive use of short videos is taken as the core independent variable, mental health as the dependent variable, and factors at the individual level (such as gender, age, household registration, monthly family income, family structure, etc.), psychological level (such as coping style, emotion regulation, social support, etc.) and behavioral level (such as exercise frequency, academic performance) are included as potential statistical mediators or moderators. Firstly, the study adopts random forest (RF) and linear support vector regression (SVR) models, treating all individual, psychological and behavioral level variables as well as the independent variable as features, with mental health as the target, to screen out the core factors with significant predictive power for mental health. On this basis, a path analysis (PA) is further constructed to systematically evaluate potential statistical pathways linking excessive use of short videos with mental health. Since the survey reports of World Health Organization (2020, 2024) and large-scale cohort data (Kelly et al., 2018) all show that excessive use of short videos or social media is an important predictive correlate of mental health problems in teenagers, and the research focus on the influence mechanism is also more concentrated on psychological factors, Hypothesis 1 is proposed: Excessive use of short videos and psychological factors (such as coping style, emotion regulation, social support) are key features for predicting the mental health of vocational students. Based on the I-PACE model and related meta-analysis studies (Nguyen et al., 2025; Shannon et al., 2022), Hypothesis 2 is proposed: Excessive use of short videos is directly associated with the mental health of vocational students and is also indirectly associated with it through psychological factors. This study describes potential pathways linking short-video use and mental health, going beyond the simple “use–symptom” model, and provides a more refined theoretical framework for understanding the mental health of vocational students on social media. The research results may help identify candidate prevention and support targets and provide preliminary evidence for developing evidence-informed mental health support measures for vocational students.

## 2. Materials and Methods

### 2.1. Participants

In this study, a total of 8346 participants were recruited from vocational high schools in Tianjin, China. All participants provided informed consent prior to their involvement. Ethical approval for the research was obtained from the Ethics Committee of Tianjin Normal University (Approval No. 2025110302). Data quality was assessed via the inclusion of lie-detection items. Participants were excluded if they failed to respond as instructed (e.g., not selecting the “quite serious” option for designated items) or if their response discrepancies on parallel content items were ≥3 points. A total of 6096 valid datasets were retained, valid response rate of 73.0%. The participants ranged in age from 15 to 20 years (M = 16.43, SD = 1.11), with 69.7% identifying as male and 30.3% as female. Additional demographic variables are presented in Table 1.

### 2.2. Research Instruments

#### 2.2.1. Symptom Checklist-90 (SCL-90)

This study utilized the SCL-90 to assess the mental health status of vocational high school students. This scale, developed by Derogatis et al., is now one of the most widely used instruments for assessing psychological status (Derogatis et al., 1973). This scale comprises 90 items, with subscales including somatization, obsessive–compulsive symptoms, interpersonal sensitivity, depression, anxiety, hostility, phobia, paranoia, and psychoticism. Each item uses a 5-point Likert scale, where scores of 1 to 5 correspond to “none,” “mild,” “moderate,” “moderately severe,” and “severe,” respectively. A higher total score indicates more prominent psychological distress in the individual. In the present study, all subscales of this instrument demonstrated generally good reliability and validity; further details are provided in Table 2.

#### 2.2.2. Short-Video Excessive Use Scale

The Short-Video Excessive Use Scale used in this study was adapted from the Facebook Intrusion Questionnaire developed by Elphinston and Noller (2011), with the term “Facebook” replaced by “short video.” The original eight-item version showed acceptable internal consistency (Cronbach’s α = 0.79), with CFA indices of χ^2^/df = 33.55, CFI = 0.93, TLI = 0.90, RMSEA = 0.07, and SRMR = 0.04. Items 2 and 5 had standardized factor loadings below the prespecified threshold of 0.50. These two items were therefore removed.

The resulting six-item version showed slightly higher internal consistency (Cronbach’s α = 0.80) and improvements in CFI, TLI, and SRMR (CFI = 0.95, TLI = 0.92, and SRMR = 0.03). However, RMSEA increased from 0.07 to 0.08, and χ^2^/df increased from 33.55 to 36.03, indicating that item removal did not uniformly improve global model fit. Nevertheless, Items 2 and 5 were relatively weak indicators of the intended construct, as their standardized factor loadings were below 0.50, whereas all retained items met the loading criterion. Moreover, the RMSEA of 0.08 remained within the conventionally acceptable range, and χ^2^-based indices are particularly sensitive to large sample sizes. Therefore, the six-item version was retained for subsequent analyses.

#### 2.2.3. Self-Developed Scale

The present study used a self-developed questionnaire to assess participants’ coping styles, social support, and emotion regulation strategies. Given the large-scale nature of the present study, single-item measures were employed for selected variables to minimize participants’ response burden and improve response rates. Prior research has demonstrated that single-item measures exhibit satisfactory psychometric properties for psychological constructs with clear and specific conceptual definitions (Wanous et al., 1997). All items in the questionnaire were adapted from well-validated, psychometrically sound scales in their respective domains, with the most representative items selected for administration. All items were rated on a 5-point Likert scale, ranging from 1 (completely inconsistent) to 5 (completely consistent), with higher scores indicating greater prominence of the dimension-specific characteristics.

Coping styles were assessed primarily using the Simplified Coping Style Questionnaire (SCSQ), developed by Xie (1998). For the present study, two core items were extracted from the scale to measure individuals’ tendencies toward positive and negative coping, respectively. Specifically, the item assessing positive coping was: “When encountering difficulties, I actively seek help from friends or family”; the item assessing negative coping was: “When facing stress, I often relieve it by avoiding the situation”.

Emotion regulation strategies were assessed with reference to the Emotion Regulation Questionnaire (ERQ), developed by Gross and John (2003). Drawing on Gross’s process model of emotion regulation, representative items were selected to assess cognitive reappraisal and expressive suppression, respectively. The cognitive reappraisal item read: “When I feel down, I attempt to reframe the situation to improve my mood”; the expressive suppression item read: “Even when I feel sad, I try to avoid showing it in front of others”.

The measurement of social support was adapted from the Perceived Social Support Scale (PSSS) developed by Zimet et al. (1988). For the purpose of this study, two key subscale indicators were selected to operationalize social support: Family support was assessed via the item: “My family provides me with emotional support when I am in need”; Friend support was assessed via the item: “When I face difficulties, there is at least one or two friends who are willing to listen to me”. Responses to these two items were aggregated to generate a composite social support score. The two items were moderately positively correlated (r = 0.48, *p* < 0.001), indicating that family and friend support were related but not interchangeable. Their scores were aggregated to form a brief composite indicator of perceived social support.

To provide preliminary criterion-related and convergent validity evidence for these brief indicators, we examined their Pearson correlations with the SCL-90 total score and with theoretically related constructs. Social support (*r* = −0.430, *p* < 0.001), positive coping (*r* = −0.381, *p* < 0.001), and cognitive reappraisal (*r* = −0.371, *p* < 0.001) were negatively correlated with psychological distress, whereas negative coping (*r* = 0.311, *p* < 0.001) and expressive suppression (*r* = 0.127, *p* < 0.001) were positively correlated with psychological distress. Consistent with the expected pattern of convergent associations, positive coping was positively correlated with social support (*r* = 0.565, *p* < 0.001) and cognitive reappraisal (*r* = 0.424, *p* < 0.001); social support was also positively correlated with cognitive reappraisal (*r* = 0.493, *p* < 0.001), and negative coping was positively correlated with expressive suppression (*r* = 0.168, *p* < 0.001). Spearman correlations showed the same overall pattern.

### 2.3. Data Analysis

#### 2.3.1. Reliability and Validity Analysis

Reliability and validity of the scales described above were examined using Mplus (v8.3) and SPSS (29.0).

#### 2.3.2. Machine Learning Modeling

Data cleaning, modeling, and evaluation were performed in a Python 3.11 environment using the Scikit-learn library. For ten continuous mental health indicators—including somatization, obsessive–compulsive symptoms, interpersonal sensitivity, depression, anxiety, hostility, phobia, paranoia, psychoticism and total score—predictive models were constructed separately. To ensure the robustness of the findings, two independent models—namely, RF (a nonlinear ensemble algorithm) and SVR (with a linear kernel)—were developed for cross-validation. If both models converge on similar key features, these features can be regarded as highly credible markers.

(1) Data Preprocessing

Predictor variables were categorized into three types: nominal variables (gender, household registration status, family structure), ordinal variables (monthly household income, exercise frequency, self-reported academic performance, positive coping, negative coping, social support, cognitive reappraisal, expressive suppression), and continuous variables (age, problematic short-video use).

Nominal variables were converted into dummy variables via one-hot encoding. For the standardization of ordinal and continuous variables, differentiated strategies were employed based on model-specific characteristics: Linear SVR applied Z-score standardization to both ordinal and continuous variables to accelerate convergence speed and improve numerical stability; RF retained the original numerical scales of the variables.

To assess potential multicollinearity, the study calculated the Variance Inflation Factor (VIF) for ordinal and continuous variables. Results indicated that the VIF values of all included variables were less than 5, suggesting no severe multicollinearity issues.

(2) Model Construction and Optimization

This study employed two algorithms—RF and Linear SVR—for model development. The dataset was randomly split into a training set and a test set at a ratio of 80:20, with a fixed random seed (Random State = 42). Model hyperparameter optimization was performed on the training set using random search coupled with 5-fold cross-validation. The search space for RF encompassed hyperparameters including the number of decision trees (n_estimators), maximum tree depth (max_depth), minimum samples required to split an internal node (min_samples_split), minimum samples required at a leaf node (min_samples_leaf), and maximum number of features considered for splitting (max_features). The search space for Linear SVR encompassed hyperparameters including the regularization coefficient (C), the epsilon-insensitive loss parameter (epsilon), and the intercept fitting flag (fit_intercept). The maximum number of iterations was set to 50,000 to ensure algorithmic convergence.

(3) Model Evaluation and Feature Importance

To assess model performance and robustness, the present study employed a multi-dimensional evaluation framework. Specifically, for the evaluation of generalization ability, the coefficient of determination (*R*^2^) and root mean square error (RMSE) were computed on the independent test set. Subsequently, for stability assessment, 10-fold cross-validation was performed on the optimal model derived from hyperparameter tuning within the training set, and the mean and standard deviation of the *R*^2^ were reported.

To examine the relative contribution of each predictor variable to mental health outcomes, this study employed a permutation-based feature importance method. Specifically, within the test set, each feature was sequentially permuted (randomly shuffled) while holding all other features constant, and the corresponding decrease in the model’s coefficient of determination *R*^2^ was calculated. This process was repeated 5 times, with the average decrease in *R*^2^ serving as the metric to quantify the importance of each feature.

#### 2.3.3. Path Analysis

To further examine potential pathways in the association between machine learning-identified key features and mental health outcomes, the present study first extracted the top 4 features. Subsequently, guided by an established theoretical framework, a mediation model titled “Excessive Short-Video Use → Psychological Factors → Mental Health Outcomes” was constructed. The proposed mediation model was estimated via Mplus v8.3. The significance of statistical indirect effects was tested using the bias-corrected Bootstrap method, with 1000 resamples and a 95% confidence interval (CI) computed. An indirect effect was deemed statistically significant if the 95% CI did not include zero.

Because the machine learning feature selection procedure and the primary path analysis were conducted within the same overall sample, we conducted a supplementary internal split-sample sensitivity analysis to assess potential selection-induced optimism. The 6096 participants were divided into a discovery sample (70%, *n* = 4267) and a non-overlapping confirmation sample (30%, *n* = 1829), stratified by gender, urban/rural household registration status, and quintiles of the SCL-90 total score. RF and linear SVR models predicting the SCL-90 total score were tuned exclusively within the discovery sample, and the same four core predictors were identified. After the theory-specified path structure had been fixed, nuisance models fitted in the discovery sample were applied to the confirmation sample to generate standardized residuals. The path model was then estimated using these confirmation sample residuals with 1000 bias-corrected bootstrap draws.

## 3. Results

### 3.1. Key Features Associated with Mental Health Outcomes

#### 3.1.1. Model Parameter Tuning and Optimal Hyperparameters

Using the training set data, optimal hyperparameterized models for each prediction target were identified via 5-fold cross-validation and a random search strategy. For the RF model, the optimal hyperparameter combinations were highly consistent across all mental health indicators (the nine subscales and the total score), with specific settings detailed below: n_estimators = 400, max_depth = 10, min_samples_split = 5, min_samples_leaf = 2, max_features = ‘sqrt’.

For the linear SVR model, optimal hyperparameters exhibited slight variability across different prediction targets. Specifically, for the prediction targets of somatization, depression, anxiety, hostility, paranoia, and total score, the optimal hyperparameters are as follows: C = 50.0, epsilon = 0.2, fit_intercept = True. For the remaining four mental health indicators, the optimal regularization parameter (C = 10.0) was smaller, and no intercept term was included in the model specification. This finding suggests that the complexity of linear decision boundaries varies across different psychological dimensions.

#### 3.1.2. Model Performance Assessment and Cross-Model Comparison

**Figure 1:**
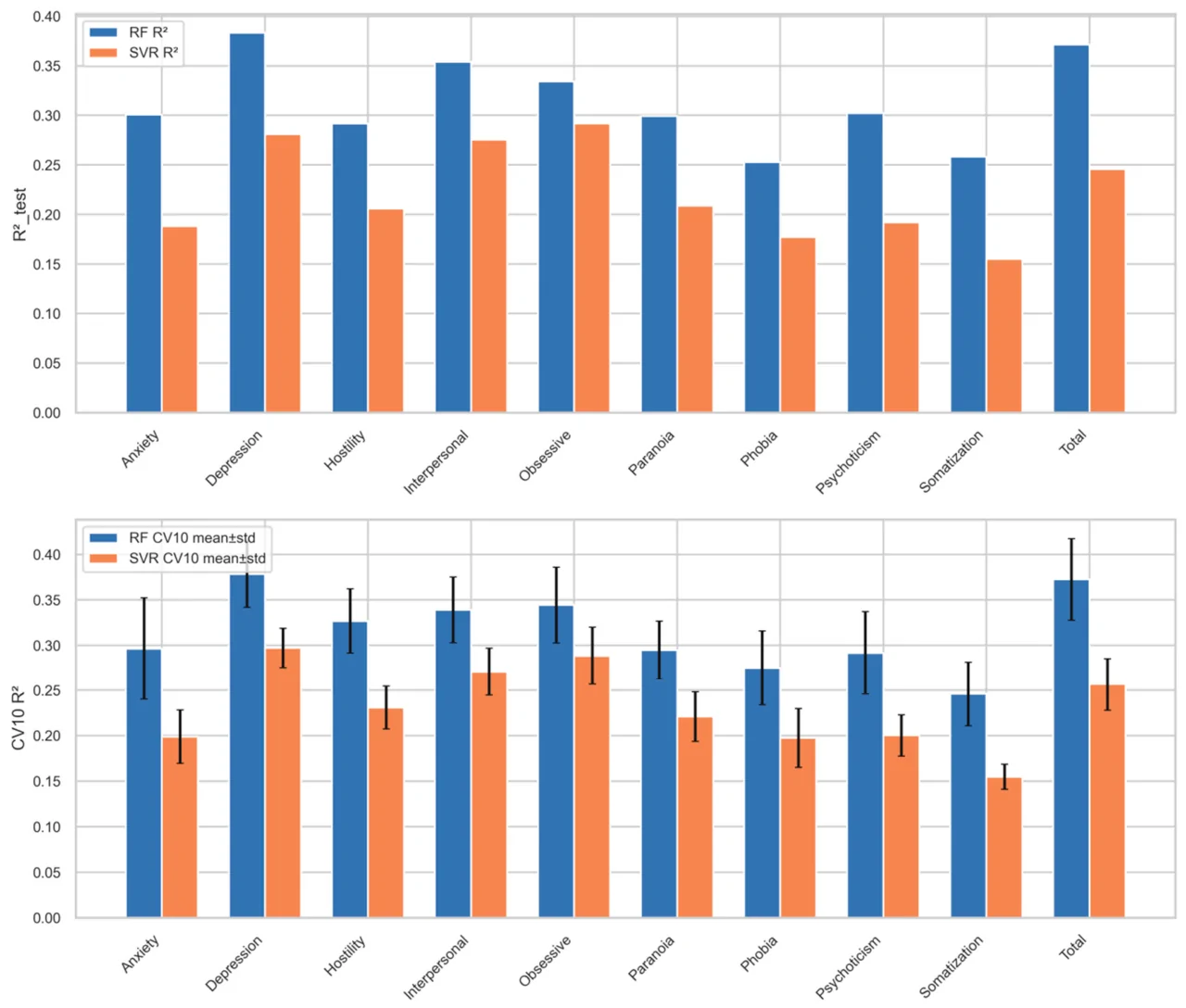
Performance Assessment and Cross-Model Comparison of RF and Linear SVR Models. Note: The top figure shows the model evaluation results of the test set. The bottom figure shows the evaluation results of the ten-fold cross-validation of the training set.

The RF model outperformed the linear SVR model across all mental health outcomes, as evidenced by a higher coefficient of determination *R*^2^ and a lower RMSE. Figure 1 presents the predictive performance (as measured by *R*^2^) of the two models across the independent test set and their average performance from ten-fold cross-validation (10-fold CV) on the training set. Taking depression as an illustrative example: the RF model explained 38.3% of the variance in the test set (RMSE = 0.58), which was significantly higher than the 28.1% variance explained by the linear SVR model (RMSE = 0.63). For the total score of mental health, the RF model achieved a test-set *R*^2^ of 0.37 (RMSE = 43.23), compared to an LSVR test-set *R*^2^ of 0.25 (RMSE = 47.35). These results indicate that the RF model demonstrates valid predictive capacity for individuals’ overall mental health levels. Additionally, results from the ten-fold cross-validation (10-fold CV) were highly consistent with those from the independent test set, with a small standard deviation (ranging from 0.014 to 0.056). This indicates that neither model exhibited significant overfitting, demonstrating robust generalization ability.

#### 3.1.3. Feature Importance Analysis

**Figure 2:**
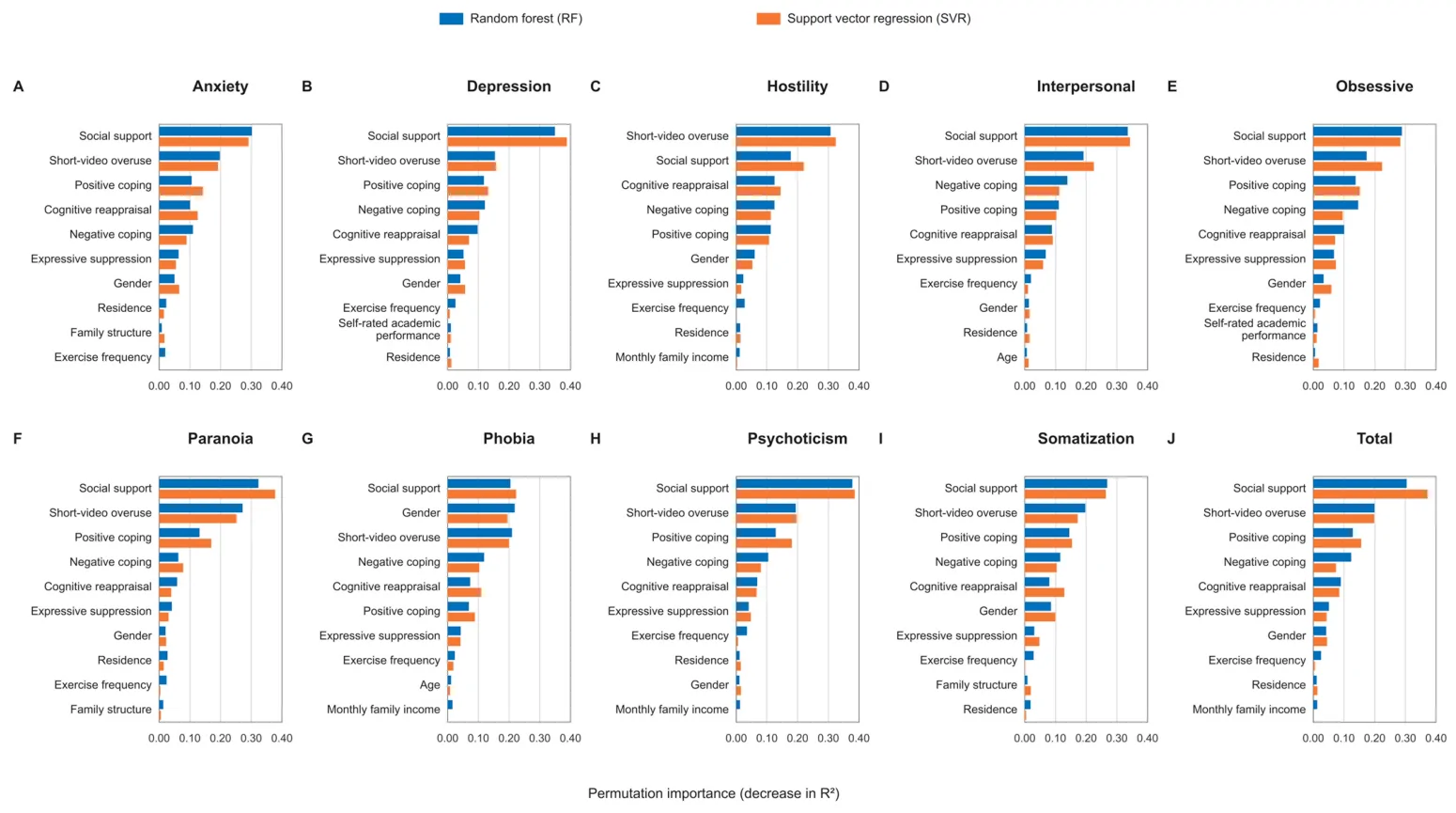
Top 10 Features Identified via Permutation Feature Importance Analysis.

For the total mental health score, the top four features identified by both the RF and SVR models were largely consistent: social support, excessive short-video use, positive coping, and negative coping. These results indicate that the four variables serve as key predictors of mental health outcomes. In contrast, demographic variables (e.g., household registration status, monthly family income), exercise frequency, and self-reported academic performance exhibited relatively lower importance rankings. The ranking pattern of the aforementioned features also exhibited high stability across subscales including depression, interpersonal, obsession, paranoia, psychoticism, and somatization with the results presented in Figure 2. These findings further validate the robustness of the four key features identified earlier.

### 3.2. Potential Pathways Linking Excessive Short-Video Use to Mental Health Outcomes

#### 3.2.1. Data Preprocessing

Prior to constructing the PA, this study implemented a two-step preprocessing procedure to account for confounding effects of non-core features and test the functional form of relationships between core variables—thereby ensuring the accuracy and robustness of subsequent path analyses.

(1) Statistical Control for Non-Critical Features

To rigorously control for potential nonlinear effects of non-critical features and accurately estimate the net associations between the four core features and mental health outcomes, this study employed a RF-based double cross-fitting residualization strategy. Specifically, within a 10-fold cross-validation framework, a RF model was used to estimate the conditional expectations of mental health outcomes and core features given non-critical features (gender, household registration, family structure, monthly household income, exercise frequency, self-reported academic performance, cognitive reappraisal, expressive suppression, and age). Residuals—representing the confounding-adjusted values—were derived by computing the difference between observed and predicted values on independent test folds, and these residuals were retained for subsequent analyses. Model parameters for the RF were set to the optimal values identified in the preceding analyses.

(2) Analysis of Net Associations Between Core Features and Mental Health Outcomes

To examine the functional relationships between core features and mental health outcomes after accounting for confounding effects of non-critical features, we constructed linear models (y~x) and quadratic polynomial models (y~x + x^2^) for each “single feature–single target” pair in a Python 3.11 environment using the scikit-learn library. Specifically, the four core features served as predictors, and the ten mental health outcomes as response variables. Five-fold cross-validation was employed to assess model generalization performance and mitigate overfitting, with the mean coefficient of determination *R*^2^ used as the metric for evaluating model explanatory power. We computed the difference in cross-validated *R*^2^ values between the quadratic and linear models, and performed a paired *t*-test to examine whether the inclusion of the quadratic term significantly enhanced model generalization performance. A quadratic model can be deemed superior only if the *t*-test yields a significant result and the change in R-squared (Δ*R*^2^) is greater than 0. Results indicated that across all tested predictor–response pairs, adding the quadratic term did not lead to a statistically significant improvement in generalization performance (see Table 3), suggesting that the relationships between core features and mental health outcomes are more likely to be linear, justifying the subsequent path analysis.

#### 3.2.2. Path Analysis

(1) Hypothesized Model

**Figure 3:**
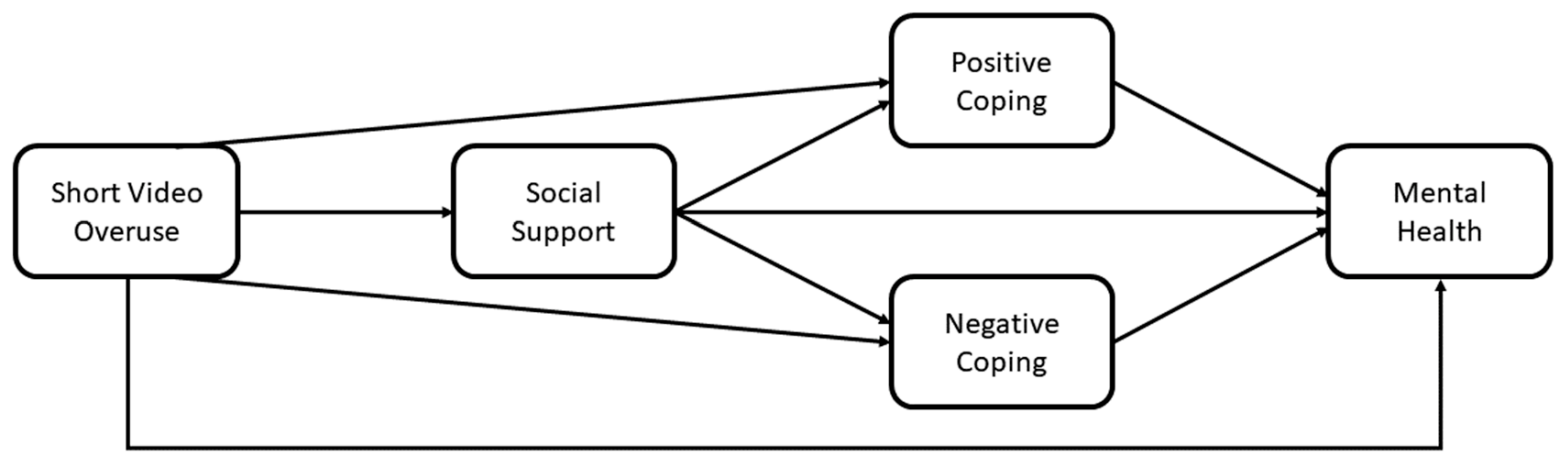
Hypothesized Model of Core Features and Mental Health Outcomes.

As evidenced by World Health Organization (2020, 2024) surveys and prior empirical research (Kelly et al., 2018; Nguyen et al., 2025), excessive short-video use has been associated with adolescent mental health. The Social Displacement Theory and Stress Coping Theory respectively provide theoretical grounding for the mediating roles of social support and stress coping in this relationship. Specifically, the Social Displacement Theory posits that excessive immersion in virtual environments diminishes in-person social connections with family and friends, thereby reducing individuals’ perceived real-world social support (Al-Kandari & Al-Sejari, 2021). By compromising social support—a crucial protective factor for mental health—excessive short-video use diminishes this buffer, which may increase individuals’ vulnerability to psychological distress. Rooted in Stress Coping Theory, negative coping strategies do not address the root causes of stressors; instead, they perpetuate stress accumulation, and may be associated with psychological maladjustment. In contrast, positive coping strategies serve as a significant buffer against the adverse effects of external stress on mental health (Lazarus & Folkman, 1984). Empirical studies have documented a significant correlation between adolescent mobile phone addiction and coping styles (Lu et al., 2021), with excessive short-video use frequently conceptualized as an avoidant coping strategy (Y. Liu et al., 2021). Furthermore, Conservation of Resources (COR) Theory posits that excessive short-video engagement initially depletes individuals’ social resources, triggering a resource loss spiral (Hobfoll, 1989). Specifically, individuals with limited social support lack the requisite resources to employ adaptive coping strategies when confronting stressors, compelling them to rely on maladaptive coping instead. This sustained resource depletion may be associated with poorer mental health. Grounded in these theoretical frameworks and empirical findings, the present study proposes the following hypothetical model (see Figure 3).

(2) Model Fit Evaluation Results

To comprehensively investigate the effects of the four core features on both the overall level and specific dimensions of mental health, this study first constructed a PA with the total score of the SCL-90 as the dependent variable to test the hypothesized model. Subsequently, to examine the robustness and generalizability of this influence mechanism across distinct domains of mental health, we built nine additional parallel PAs, each specifying one of the nine subscales of the SCL-90 as the dependent variable.

Exploratory factor analysis was performed on the variables involved in each of the 10 path analysis individually using the Harman single-factor test method. The results indicated that the variance explained by the first common factor for each model was less than 40%, suggesting no evidence of severe common method bias based on this diagnostic test. Furthermore, correlation analysis was performed on the variables included in these 10 path analyses, revealing that significant correlations existed between all pairs of variables (*p*s < 0.001).

The fit indices of the PA, in which excessive short-video use serves as the independent variable, social support, positive coping, and negative coping act as the mediating variables, and the total mental health score as the dependent variable, are presented below: χ^2^/df = 5.86, CFI = 0.998, TLI = 0.99, RMSEA = 0.03, SRMR = 0.01. The other nine models constructed by taking each of the nine factors of the SCL-90 as the dependent variable exhibited fit indices that were highly consistent with those of the total mental health score model.

**Figure 4:**
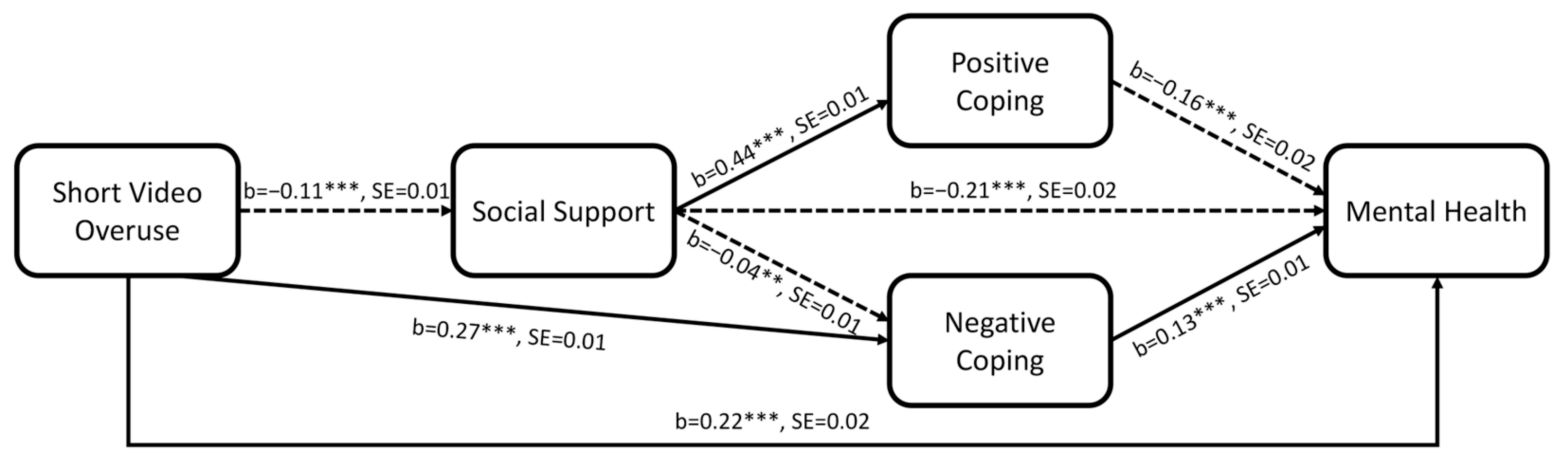
Path Model of Total Mental Health Score. Note: This path model was also validated across all nine subscales of the SCL-90; solid lines indicate positive predictive effects, and dashed lines indicate negative predictive effects; *** = *p* < 0.001, and ** = *p* < 0.01.

A bias-corrected nonparametric percentile Bootstrap method (*n* = 1000) was employed to examine the mediating effects of social support, positive coping, and negative coping in the relationship between excessive short-video use and mental health. The results are presented in Figure 4. In the model using the total mental health score as the outcome variable, excessive short-video use was associated with mental health problems through lower social support (effect = 0.023, 95% CI = [0.017, 0.031]); excessive short-video use was associated with greater mental health problems through higher negative coping strategies (effect = 0.035, 95% CI = [0.028, 0.042]); excessive short-video use was associated with lower social support, which was in turn associated with reduced positive coping and, ultimately, with greater psychological distress (effect = 0.007, 95% CI = [0.005, 0.010]); after controlling for the three aforementioned mediating pathways, the direct effect of excessive short-video use on mental health remained statistically significant (effect = 0.221, 95% CI = [0.189, 0.253]). This indicates that social support, positive coping, and negative coping showed small partial statistical mediation in the relationship between excessive short-video use and mental health. Notably, although all individual path coefficients along the chain pathway “excessive short-video use → social support → negative coping → mental health” are statistically significant, the confidence interval for the indirect effect derived from the Bootstrap analysis includes zero (95% CI [0.000, 0.001]). Thus, this chain mediating effect is not statistically significant. Furthermore, in the models with the nine subscales of the SCL-90 as dependent variables, the aforementioned simple mediating effects and chain mediating effects remained robust, as presented in Tables S1 and S2 in the Supplementary Materials.

#### 3.2.3. Internal Split-Sample Sensitivity Analysis

The discovery and confirmation samples showed minimal differences across the examined variables; the largest absolute standardized mean difference or Cramér’s V was 0.066. In the discovery sample, consensus feature ranking across RF and linear SVR for the SCL-90 total score identified the same four core predictors as the primary analysis: social support, excessive short-video use, positive coping, and negative coping. In the confirmation sample, the direct association between excessive short-video use and the SCL-90 total score was 0.222 (95% CI [0.173, 0.280]). The indirect associations through lower social support (effect = 0.019, 95% CI [0.009, 0.032]), higher negative coping (effect = 0.036, 95% CI [0.023, 0.054]), and lower social support followed by lower positive coping (effect = 0.005, 95% CI [0.003, 0.010]) also remained statistically detectable. These three indirect associations and the direct association were also observed for all nine SCL-90 subscales. Detailed results are provided in the Supplementary Materials (Tables S3 and S4, Figure S1).

## 4. Discussion

This study aimed to investigate the associations between excessive short-video use and the mental health of vocational students and to examine potential pathways linking them. By constructing an integrated analytical framework that integrates individual, psychological, and behavioral dimensions, the following core findings were derived: (1) Both RF and linear SVR identified the same four key predictors—social support, excessive short-video use, positive coping, and negative coping—thereby providing support for Hypothesis 1. (2) Results from the PA indicated that excessive short-video use was directly associated with poorer mental health among vocational students and also showed smaller indirect associations through statistical pathways involving psychological factors such as social support and coping styles, thus supporting Hypothesis 2. It is important to note that, given the cross-sectional design, the directional paths were specified based on relevant theories, and the present analysis only examined whether this theory-guided ordering was consistent with the observed data. Therefore, the findings cannot establish causality or temporal ordering.

### 4.1. Analysis of Key Predictors of Mental Health Among Vocational Students

This study revealed that social support, excessive short-video use, positive coping, and negative coping ranked as the top four features in terms of importance when predicting the total mental health score. This ranking was stable across six subscales—depression, interpersonal sensitivity, obsessive–compulsive symptoms, paranoia, psychoticism, and somatization—indicating consistency across symptom domains within this Chinese vocational high school sample.

Social support consistently emerged as the top-ranked predictor across the majority of models for mental health indicators. This finding suggests that, in the context of predicting vocational students’ mental health, external support systems may have stronger predictive relevance than internal personal characteristics (e.g., coping styles) or behavioral habits (e.g., exercise frequency). Prior research has also established social support as a core protective factor against adverse outcomes (Cohen & Wills, 1985). For vocational students, whose self-regulatory capacities and metacognitive functioning are still developing (Steinberg, 2005), social support acts as an external resource that may provide timely support or compensation when internal coping resources are insufficient, serving as a protective resource for stress resilience. This developmental limitation is rooted in the asynchronous maturation of the prefrontal cortex and the limbic system during adolescence, which constrains autonomous emotion regulation and heightens reliance on external regulatory resources (Sahi et al., 2023). This aligns with the stress-buffering hypothesis (Ozbay et al., 2007), which posits that social support mitigates the negative effects of stress by enhancing coping resources. Furthermore, social support is derived not only from family but also crucially from peer networks within the campus environment. Vocational students are in a developmental stage characterized by a transition from family attachment to peer attachment (Gorrese & Ruggieri, 2012), during which sensitivity to peer evaluation and the need for social belonging become increasingly salient (Rodman et al., 2023). Notably, most vocational high school students enter, at around the age of 15, a learning environment organized around major-based specialization and collaborative practical training. This early exposure to a vocationally oriented context may make peer relationships particularly consequential for their mental health (Cui et al., 2026). Peer emotional co-regulation mechanisms have been shown to be more effective than adult-led interventions in alleviating anxiety and hostility (Borowski & Zeman, 2025; Hostinar et al., 2014). Given that vocational education settings often foster close cohort-based and workshop-based peer interactions, such contexts may offer unique opportunities for cultivating supportive peer bonds. Thus, fostering and strengthening peer support networks may be a useful strategy for reducing psychological symptoms and supporting vocational students with complex mental health needs.

Excessive short-video use consistently ranked among the top predictors across all mental health indicator models, even occupying the first position in the hostility subscale model. Its explanatory power far surpassed that of other behavioral-level features, such as exercise frequency and academic performance. This finding suggests that, among contemporary vocational students, media usage habits may show stronger associations with mental health than some traditional behavioral indicators. Schmidt-Persson et al. (2024) identified screen media use as a critical risk factor for the mental health of children and adolescents. Similarly, the usage patterns of short videos and social media among adolescents are closely linked to their mental health outcomes (M. Liu et al., 2024). Short-video browsing may not be merely a time-consuming activity; it may function as a digital stressor. Algorithm-driven fragmented information streams, characterized by extremely high reward feedback frequencies, deliver immediate sensory stimulation while potentially being associated with weaker impulse control and cognitive functioning (Arouch et al., 2025; Firth et al., 2019). During adolescence, limbic systems involved in reward processing mature earlier than the prefrontal systems responsible for cognitive control (Steinberg, 2005). This dual-systems imbalance makes adolescents more sensitive to immediate rewards and more readily attracted to the high-frequency feedback provided by short videos. Over prolonged periods, this may be associated with disruptions in emotional regulation processes (Gao et al., 2025) and lower the threshold of psychological defense mechanisms. Thus, short-video usage could be considered in school-based assessment frameworks for vocational education populations as a behavioral indicator relevant to psychological risk screening (Insel, 2017; Yang et al., 2025).

Coping styles refer to internal regulatory strategies through which individuals engage in cognitive restructuring and behavioral adaptation in response to stressful contexts, and are widely recognized as core predictors of mental health (Compas et al., 2017). The present study similarly found that both positive and negative coping styles exhibit substantial explanatory power for the mental health outcomes of vocational students. For vocational students undergoing psychosocial transition, coping styles function not only as an extension of their personality traits but also as a critical determinant of their ability to sustain psychological resilience when confronted with stressors (Campbell-Sills et al., 2006).

Furthermore, by comparing feature importance rankings across different mental health subscales, this study identified subtle heterogeneities in predictive patterns. Specifically, in the prediction models for anxiety and hostility, cognitive reappraisal emerged among the top four most important predictors. This finding suggests that for issues characterized by high emotional explosiveness (e.g., hostility) or future-oriented anxiety, individuals’ emotional regulation capacity may play a more critical role than general coping styles alone. In the prediction model for the phobia subscale, the importance of gender increased significantly—likely reflecting higher susceptibility to specific phobias among females (Wardenaar et al., 2017). However, the present sample was predominantly male (69.7%). This finding therefore requires replication in samples with a more balanced gender composition and suggests that predictors of different psychological symptoms may vary by individual characteristics such as gender.

Notably, despite these minor variations in feature rankings across distinct psychological symptoms, social support, excessive short-video use, positive coping, and negative coping—consistently identified as core predictive variables—all remained within the top six positions in terms of importance across all mental health models.

### 4.2. Potential Pathways Linking Excessive Short-Video Use to Vocational Students’ Mental Health: A Mediating Analysis

Using PA, this study identified several statistically detectable indirect associations linking excessive short-video use to vocational students’ mental health problems, with a consistent overall pattern across all nine SCL-90 dimensions. However, in the total-score model, these indirect associations were small (effects = 0.007–0.035, each accounting for 2.6–12.0% of the total association), and their statistical detectability in this large sample should not be equated with practical importance. Thus, the proposed mediating pathways accounted for only a limited part of the total association.

The findings revealed that excessive short-video use showed a significant direct association with vocational students’ mental health problems, with most of the total association accounted for by the direct pathway (e.g., the direct statistical association accounted for 76.7% of the total association when the dependent variable was the total mental health score). This indicates that even when excluding mediating factors such as social interaction patterns or coping styles, excessive digital media consumption itself remained closely associated with poorer mental health. This observation aligns with the World Health Organization’s warnings regarding digital health risks (World Health Organization, 2020, 2024). Vocational students often confront heightened pressure related to vocational skill acquisition and social stigma. On the one hand, whereas general high school education is primarily oriented toward the single goal of university admission, vocational education requires students to undergo the transition from students to prospective practitioners during adolescence—a developmental period in which cognitive capacities and identity are still rapidly evolving (Steinberg, 2005)—and to gradually develop a vocational identity; namely, a clear and stable sense of themselves in relation to a future occupation. Because practical training repeatedly subjects students to evaluations based on procedural requirements and performance standards while their skills are still developing, such frequent and highly visible assessments of competence may intensify vocational frustration and self-doubt (Cao & Han, 2024). On the other hand, within a Chinese social context that tends to privilege general education over vocational education, vocational students are frequently labeled as “academic failures” and subjected to other negative stereotypes. Such public stigma may be internalized as self-devaluation and a negative self-identity, thereby becoming a persistent source of psychosocial stress (Leng et al., 2025; Wang et al., 2022). The immediate rewards provided by short videos offer a low-cost dopamine-based compensatory mechanism for these stressors. However, as previously noted, prolonged exposure to high-frequency, high-intensity, and fragmented information from short videos may be associated with poorer cognitive functioning and emotion regulation, potentially contributing to poorer mental health (Arouch et al., 2025; Gao et al., 2025).

Excessive short-video use was significantly associated with mental health problems through greater negative coping. Across all PAs for mental health indicators, this mediating pathway exhibited the highest proportion of the indirect effects, although its absolute effect size remained modest. This finding aligns with the proposition of Liu et al. that excessive short-video use itself constitutes an avoidant coping strategy (Y. Liu et al., 2021). Drawing on stress and coping theory (Lazarus & Folkman, 1984), vocational students often turn to short videos as a temporary refuge to escape academic or employment-related stressors. However, empirical evidence indicates that this strategy, when accompanied by unresolved real-life conflicts, may be associated with greater psychological distress (Compas et al., 2017). For vocational students in the formative stage of their vocational identity, long-term reliance on such negative coping strategies may intensify their sense of powerlessness in real-life contexts, potentially placing them in a cycle of stress, avoidance, and amplified stress. This is especially true when avoidance behaviors hinder the actual acquisition of vocational skills, which may further undermine their still-developing vocational identity, creating a vicious cycle in which skill-related setbacks and identity-related anxiety mutually reinforce each other.

Social support also plays a crucial role in the mediating path. First, excessive use of short videos was associated with poor mental health through lower social support. This result supports the social displacement hypothesis (Al-Kandari & Al-Sejari, 2021). The pseudo-intimacy inherent in the digital social interactions of vocational students may displace authentic support from friends and family, potentially limiting access to social support—a critical psychological buffer—during their pivotal developmental stage (Ozbay et al., 2007). Although this indirect association was smaller than the pathway through negative coping, it remained statistically consistent and may represent a modest pathway associated with poorer mental health. Second, the chain mediating pathway of “social support → positive coping” is also statistically significant, although its effect size is small. This finding indicates that insufficient social support is associated not only with poorer health but also with fewer psychological resources for activating positive coping strategies (e.g., seeking advice, engaging in optimistic thinking). This pathway precisely aligns with the resource depletion spiral posited by the Conservation of Resources (COR) Theory (Hobfoll, 1989, 2001): individuals who experience resource loss become not only more susceptible to further losses but also face losses that escalate in speed and severity. Excessive short-video use may first be associated with reduced social resources of vocational students, which in turn may be linked to lower use of positive coping strategies when confronting stressors and to greater psychological difficulties. Given the small indirect effect, this pathway should be interpreted cautiously. It suggests that excessive short-video use may be linked to fewer positive psychological resources and less adaptive coping capacity.

Notably, while the serial mediating pathway excessive short-video use → social support → negative coping → mental health was not statistically significant, this non-significant result should be interpreted cautiously and suggests that the association between excessive short-video use and vocational students’ mental health may be more consistently reflected in lower positive resources (social support and positive coping) and higher negative coping, rather than in a robust serial pathway from social support to negative coping.

### 4.3. Educational Support and Practical Implications for Vocational Education

This study provides preliminary implications for mental health support in vocational education settings. Support strategies should not be limited to reducing screen time or restricting smartphone use but should also emphasize digital and social media literacy. Given that socially oriented forms of engagement and a greater sense of control over social media use are associated with better well-being (Finserås et al., 2025; Parry & Coetzee, 2025), schools could help students distinguish beneficial from excessive use, understand how algorithmic recommendations shape their behavior, and develop more purposeful and self-regulated media habits. For students showing signs of excessive use, schools could strengthen real-world social support through group activities and support from peers and families, together with targeted psychological skills training to facilitate a shift from avoidant coping to active problem-solving.

Further, this study revealed that cognitive reappraisal holds a prominent role in predicting anxiety and hostility, while gender exhibits an increased weighting in the phobia factor. This finding underscores the need for highly adaptive intervention frameworks. For students with pronounced anxiety tendencies, support programs may prioritize emotional regulation training to enhance their ability to reframe stressors. For those with elevated phobia, gender-responsive personalized stress desensitization protocols could be considered, to address the distinct manifestations of fear across genders. These targeted approaches may help mental health support better align with the heterogeneous needs of vocational students with diverse psychological and demographic profiles.

### 4.4. Research Limitations and Future Directions

While this study represents a methodological advance by integrating machine learning and path analysis, it is not without limitations. First, the cross-sectional design precludes definitive inferences about causal temporality. Future research should employ longitudinal cohort studies to test the temporal ordering between excessive short-video use and poorer mental health—particularly whether the former precedes and predicts the latter, or if bidirectional effects exist. Second, the measurement of several core variables in this study has important limitations. Social support was assessed with two items, while coping and emotion regulation strategies were assessed with single items. Although their correlations followed theoretically expected patterns, the path analysis treated these indicators as error-free and could not account for measurement error, potentially biasing the estimated associations. In addition, excessive short-video use was measured using an adapted Facebook Intrusion Questionnaire, whose construct coverage and equivalence to the original measure have not been independently established. These findings should therefore be considered preliminary. Future studies should use validated multi-item instruments, measures specifically developed for excessive short-video use, and latent-variable models that account for measurement error. Third, although the current analysis included 13 features (encompassing social support and coping styles), several potential mediating or moderating variables remain unexamined, such as sleep quality (a well-documented correlate of both digital media use and mental health) and algorithmic recommendation mechanisms (which may amplify usage through personalized content exposure). Future research should expand the analytical framework to incorporate these understudied variables, as well as other individual (e.g., impulsivity traits) factors, to develop a more holistic mental health risk prediction model for vocational students. Finally, the generalizability of the findings may be limited by both sample composition and context. The sample was predominantly male (69.7%) and had rural household registration (72.9%), which may limit generalizability to female students and those with urban household registration. Moreover, patterns of short-video engagement and experiences of vocational education are shaped by cultural and socio-geographical contexts. Because the present study was conducted among vocational high school students in China, caution is warranted when generalizing the findings to other populations, countries, regions, and vocational education systems.

## 5. Conclusions

This study developed an integrated analytical framework spanning individual, psychological, and behavioral dimensions to systematically investigate associations between excessive short-video use and vocational students’ mental health and the potential pathways linking them. The key findings are as follows: (1) Social support, excessive short-video use, positive coping, and negative coping emerge as core predictors of vocational students’ mental health, collectively explaining a significant portion of variance in mental health outcomes. (2) Excessive short-video use showed a dual association with vocational students’ mental health: it was directly associated with poorer psychological well-being and indirectly associated with mental health through multiple, generally small statistical mediating pathways involving social support and coping styles. Given the cross-sectional design and measurement limitations, these findings should be considered preliminary associations requiring replication with validated multi-item measures.

## Figures and Tables

**Table 1:** Demographic Profile of the Participants.

Distribution Characteristics	Sample Size	Percentage	Distributional Characteristics	Sample Size	Percentage
Gender			Household registration location		
Male	4246	69.7%	Urban area	1652	27.1%
Female	1850	30.3%	Rural area	4444	72.9%
Monthly household income			Family structure		
≤2000 yuan	354	5.8%	Two-parent family	4928	80.8%
2001–5000 yuan	1867	30.6%	Single-parent family	709	11.6%
5001–10,000 yuan	2580	42.3%	Reconstituted family	299	4.9%
10,001–20,000 yuan	932	15.3%	Grandparent-headed household care	76	1.2%
20,001–30,000 yuan	182	3.0%	Other	84	1.4%
≥30,000 yuan	181	3.0%	Self-reported academic performance		
Exercise frequency			Poor	824	13.5%
Almost no exercise	1096	18.0%	Average	3208	52.6%
1–2 times per week	3584	58.8%	Good	1476	24.2%
≥3 times per week	1416	23.2%	Excellent	588	9.6%

**Table 2:** Reliability and Validity of Each Factor in the SCL-90.

Factor	Cronbach’s α	χ^2^/df	*p*	CFI	TLI	RMSEA	SRMR
Somatization	0.92	14.94	*p* < 0.001	0.94	0.93	0.05	0.04
Obsessive	0.90	34.11	*p* < 0.001	0.93	0.91	0.07	0.04
Interpersonal	0.90	23.83	*p* < 0.001	0.95	0.94	0.06	0.03
Depression	0.94	16.93	*p* < 0.001	0.95	0.94	0.05	0.03
Anxiety	0.92	19.10	*p* < 0.001	0.95	0.94	0.05	0.03
Hostility	0.87	52.59	*p* < 0.001	0.92	0.87	0.09	0.04
Phobia	0.86	13.43	*p* < 0.001	0.97	0.95	0.05	0.03
Paranoia	0.84	9.78	*p* < 0.001	0.98	0.97	0.04	0.02
Psychoticism	0.89	6.14	*p* < 0.001	0.98	0.97	0.03	0.02

Note: χ^2^/df = relative chi-square; CFI = Comparative Fit Index; TLI = Tucker–Lewis Index; RMSEA = Root Mean Square Error of Approximation; SRMR = Standardized Root Mean Square Residual. According to standard criteria, CFI and TLI values ≥ 0.95, and RMSEA and SRMR values ≤ 0.08, indicate a good model fit. The same applies below.

**Table 3:** Comparison of Linear and Quadratic Model Fit for Predictor–Response Pairs of Core Features and Mental Health Outcomes.

Factor	Excessive Short-Video Use		Social Support		Positive Coping		Negative Coping	
	Δ*R*^2^	*p*	Δ*R*^2^	*p*	Δ*R*^2^	*p*	Δ*R*^2^	*p*
Somatization	−0.0028	0.04	0.0001	>0.05	−0.0004	>0.05	0.0007	>0.05
Obsession	0.0004	>0.05	0.0002	>0.05	−0.0001	>0.05	−0.0007	>0.05
Interpersonal	−0.0016	>0.05	−0.0001	>0.05	−0.0005	>0.05	−0.0002	>0.05
Depression	−0.0025	>0.05	0.0006	>0.05	−0.0006	0.02	0.0000	>0.05
Anxiety	−0.0021	>0.05	0.0004	>0.05	−0.0003	>0.05	0.0005	>0.05
Hostility	−0.0027	>0.05	0.0006	>0.05	−0.0002	>0.05	0.0014	>0.05
Phobia	−0.0018	>0.05	−0.0005	>0.05	−0.0006	0.03	−0.0003	>0.05
Paranoia	−0.0028	0.04	0.0000	>0.05	−0.0002	>0.05	0.0001	>0.05
Psychoticism	−0.0026	>0.05	0.0001	>0.05	−0.0005	>0.05	0.0003	>0.05
Total	−0.0028	>0.05	0.0003	>0.05	−0.0004	0.03	0.0001	>0.05

## Data Availability

The data that support the findings of this study are available from the corresponding author upon reasonable request.

## Supplementary Materials

The following supporting information can be downloaded at: https://www.mdpi.com/article/10.3390/bs16081429/s1, Table S1: Path coefficients of the path analysis for mental health indicators; Table S2: Path effects of the path analysis for mental health indicators. Table S3: Balance between the discovery and confirmation samples and discovery sample consensus feature ranking. Table S4: Confirmation sample path coefficients and direct and indirect associations. Figure S1: Comparison of direct and indirect associations in the full sample and internal confirmation sample total-score models.
