# Supplementary material for: Associations and Potential Pathways Linking Excessive Short-Video Use to Mental Health Among Vocational High School Students in China: An Integrated Analysis Based on Machine Learning and Path Analysis
Source: Behav Sci (Basel). 2026 Aug 19;16(8):1429. doi: 10.3390/bs16081429 (PMC13509611; doi:10.3390/bs16081429)
Supplement: Supplementary file 1 [file behavsci-16-01429-s001.zip › behavsci-4430427-supplementary.pdf]

Table S1 Path Coefficients of the path analysis for Mental Health Indicators

Path	Total	Somatization	Obsession	interpersonal	Depression	Anxiety	Hostility	Phobia	Paranoia	Psychoticism
SVO→MH	0.22***	0.17***	0.19***	0.21***	0.19***	0.20***	0.24***	0.19***	0.22***	0.20***
SS→MH	-0.21***	-0.16***	-0.18***	-0.22***	-0.24***	-0.19***	-0.16***	-0.15***	-0.20***	-0.21***
PC→MH	-0.16***	-0.13***	-0.16***	-0.13***	-0.15***	-0.13***	-0.14***	-0.11***	-0.15***	-0.14***
NC→MH	0.13***	0.10***	0.14***	0.13***	0.13***	0.10***	0.13***	0.13***	0.09***	0.09***
SVO→SS	-0.11***	-0.11***	-0.11***	-0.11***	-0.11***	-0.11***	-0.11***	-0.11***	-0.11***	-0.11***
SS→PC	0.44***	0.44***	0.44***	0.44***	0.44***	0.44***	0.44***	0.44***	0.44***	0.44***
SVO→PC	-0.01	-0.01	-0.01	-0.01	-0.01	-0.01	-0.01	-0.01	-0.01	-0.01
SS→NC	-0.04**	-0.04**	-0.04**	-0.04**	-0.04**	-0.04**	-0.04**	-0.04**	-0.04**	-0.04**
SVO→NC	0.27***	0.27***	0.27***	0.27***	0.27***	0.27***	0.27***	0.27***	0.27***	0.27***

Note: MH = the 10 indicators of mental health; SVO = excessive short-video use; SS = social support; PC = positive coping; NC = negative coping; *** = $p<0.001$, ** = $p<0.01$.

Table S2 Path Effects of the path analysis for Mental Health Indicators

		Total	Somatization	Obsession	Interpersonal	Depression	Anxiety	Hostility	Phobia	Paranoia	Psychoticism
SVO→MH	Direct Effect	0.221	0.174	0.191	0.208	0.194	0.195	0.236	0.189	0.219	0.201
	95%CI	[0.189, 0.253]	[0.142, 0.207]	[0.163, 0.224]	[0.179, 0.241]	[0.163, 0.225]	[0.163, 0.227]	[0.205, 0.271]	[0.157, 0.222]	[0.188, 0.251]	[0.169, 0.234]
	Proportion	0.767	0.770	0.737	0.754	0.732	0.777	0.797	0.768	0.805	0.782
SVO→SS →MH	Indirect Effect	0.023	0.017	0.020	0.024	0.026	0.020	0.017	0.016	0.022	0.023
	95%CI	[0.017, 0.031]	[0.012, 0.024]	[0.014, 0.027]	[0.018, 0.032]	[0.019, 0.034]	[0.015, 0.028]	[0.012, 0.023]	[0.011, 0.023]	[0.016, 0.030]	[0.016, 0.030]
	Proportion	0.081	0.077	0.078	0.088	0.100	0.082	0.058	0.066	0.082	0.089
SVO→NC →MH	Indirect Effect	0.035	0.027	0.038	0.036	0.036	0.027	0.034	0.034	0.023	0.024
	95%CI	[0.028, 0.042]	[0.020, 0.033]	[0.030, 0.045]	[0.029, 0.043]	[0.029, 0.043]	[0.021, 0.034]	[0.027, 0.042]	[0.026, 0.041]	[0.016, 0.030]	[0.018, 0.031]
	Proportion	0.120	0.118	0.146	0.129	0.134	0.108	0.116	0.137	0.084	0.094
SVO→PC →MH	Indirect Effect	0.001	0.001	0.001	0.001	0.001	0.001	0.001	0.001	0.001	0.001
	95%CI	[-0.002, 0.005]	[-0.002, 0.004]	[-0.002, 0.005]	[-0.002, 0.004]	[-0.002, 0.005]	[-0.002, 0.005]	[-0.002, 0.005]	[-0.002, 0.004]	[-0.002, 0.005]	[-0.002, 0.005]
	Proportion	—	—	—	—	—	—	—	—	—	—
SVO→SS →NC→MH	Indirect Effect	0.001	0.000	0.001	0.001	0.001	0.000	0.001	0.001	0.000	0.000
	95%CI	[0.000, 0.001]	[0.000, 0.001]	[0.000, 0.001]	[0.000, 0.001]	[0.000, 0.001]	[0.000, 0.001]	[0.000, 0.001]	[0.000, 0.001]	[0.000, 0.001]	[0.000, 0.001]
	Proportion	—	—	—	—	—	—	—	—	—	—
SVO→SS →PC→MH	Indirect Effect	0.007	0.006	0.008	0.006	0.007	0.006	0.006	0.005	0.007	0.007
	95%CI	[0.005, 0.010]	[0.004, 0.008]	[0.006, 0.010]	[0.004, 0.008]	[0.005, 0.010]	[0.005, 0.009]	[0.005, 0.009]	[0.003, 0.007]	[0.005, 0.009]	[0.005, 0.009]
	Proportion	0.026	0.028	0.029	0.022	0.027	0.026	0.022	0.021	0.026	0.027

Note: MH = the 10 indicators of mental health; SVO = excessive short-video use; SS = social support; PC = positive coping; NC = negative coping

Table S3. Balance between the discovery and confirmation samples and discovery-sample consensus feature ranking

Panel A. Balance between the discovery and confirmation samples

Variable	Level	Discovery (n=4,267)	Confirmation (n=1,829)	Absolute balance statistic	Metric
Age		16.436	16.428	0.007	SMD
Monthly household income		2.889	2.857	0.030	SMD
Exercise frequency		2.056	2.045	0.016	SMD
Self-rated academic performance		2.302	2.295	0.009	SMD
Positive coping		3.783	3.851	0.066	SMD
Negative coping		3.101	3.063	0.030	SMD
Social support		8.176	8.155	0.013	SMD
Cognitive reappraisal		3.911	3.946	0.035	SMD
Expressive suppression		3.885	3.851	0.033	SMD
Excessive short-video use		12.208	11.993	0.048	SMD
SCL-90 total score		133.139	132.625	0.010	SMD
Gender	Male	69.7%	69.7%	0.000	Cramer's V
Gender	Female	30.3%	30.3%	0.000	Cramer's V
Household registration	Urban	27.1%	27.1%	0.000	Cramer's V
Household registration	Rural	72.9%	72.9%	0.000	Cramer's V
Family structure	Two-parent family	81.3%	79.9%	0.041	Cramer's V
Family structure	Single-parent family	11.5%	11.9%	0.041	Cramer's V
Family structure	Reconstituted family	4.8%	5.1%	0.041	Cramer's V
Family structure	Grandparent-headed care	1.3%	1.1%	0.041	Cramer's V
Family structure	Other	1.1%	2.1%	0.041	Cramer's V

Note: SMD = standardized mean difference. Categorical variables were assessed using Cramer's V. Values are presented as means for continuous/ordinal variables and percentages for categorical levels.

Panel B. Discovery-sample consensus feature ranking (selected Top 4)

Feature	RF importance	RF rank	SVR importance	SVR rank	Consensus importance	Consensus rank
Social support	0.294	1	0.350	1	0.322	1
Excessive short-video use	0.244	2	0.196	2	0.220	2
Positive coping	0.104	5	0.161	3	0.133	3
Negative coping	0.162	3	0.079	5	0.121	4

Note: Importance values are normalized permutation importance estimates. Consensus importance is the mean of normalized RF and linear SVR importance.

Table S4. Confirmation-sample path coefficients and direct and indirect associations

Panel A. Outcome-specific path coefficients, b (SE)

Path	Total	Somatization	Obsessive	Interpersonal	Depression	Anxiety	Hostility	Phobia	Paranoia	Psychoticism
SVO -> MH	0.222 (0.028)	0.169 (0.028)	0.186 (0.028)	0.203 (0.029)	0.208 (0.028)	0.191 (0.028)	0.235 (0.028)	0.190 (0.028)	0.221 (0.029)	0.201 (0.029)
SS -> MH	-0.208 (0.030)	-0.159 (0.034)	-0.155 (0.027)	-0.207 (0.029)	-0.234 (0.030)	-0.185 (0.031)	-0.177 (0.030)	-0.142 (0.031)	-0.197 (0.030)	-0.215 (0.032)
PC -> MH	-0.132 (0.027)	-0.128 (0.030)	-0.128 (0.026)	-0.089 (0.027)	-0.123 (0.027)	-0.121 (0.028)	-0.109 (0.028)	-0.076 (0.028)	-0.124 (0.028)	-0.121 (0.029)
NC -> MH	0.126 (0.024)	0.086 (0.025)	0.145 (0.024)	0.134 (0.025)	0.131 (0.025)	0.096 (0.025)	0.123 (0.024)	0.128 (0.025)	0.085 (0.024)	0.088 (0.025)

Panel B. Common paths among the core predictors

Path	b	SE	p
SVO -> SS	-0.092	0.024	<0.001
SS -> PC	0.454	0.025	<0.001
SVO -> PC	-0.034	0.023	0.144
SS -> NC	-0.008	0.025	0.752
SVO -> NC	0.290	0.023	<0.001

Panel C. Direct, indirect, and total associations, estimate [BC bootstrap 95% CI]

Path	Total	Somatization	Obsessive	Interpersonal	Depression	Anxiety	Hostility	Phobia	Paranoia	Psychoticism
SVO -> MH (direct)	0.222 [0.173, 0.280]	0.169 [0.118, 0.222]	0.186 [0.133, 0.240]	0.203 [0.148, 0.261]	0.208 [0.156, 0.260]	0.191 [0.142, 0.248]	0.235 [0.181, 0.290]	0.190 [0.135, 0.247]	0.221 [0.167, 0.279]	0.201 [0.149, 0.260]
SVO -> SS -> MH	0.019 [0.009, 0.032]	0.015 [0.006, 0.026]	0.014 [0.007, 0.024]	0.019 [0.009, 0.031]	0.021 [0.010, 0.035]	0.017 [0.008, 0.029]	0.016 [0.007, 0.028]	0.013 [0.006, 0.023]	0.018 [0.008, 0.031]	0.020 [0.009, 0.032]
SVO -> NC -> MH	0.036 [0.023, 0.054]	0.025 [0.011, 0.040]	0.042 [0.029, 0.059]	0.039 [0.025, 0.058]	0.038 [0.024, 0.056]	0.028 [0.014, 0.044]	0.036 [0.022, 0.052]	0.037 [0.024, 0.056]	0.024 [0.010, 0.040]	0.025 [0.011, 0.042]
SVO -> PC -> MH	0.004 [-0.001, 0.013]	0.004 [-0.001, 0.013]	0.004 [-0.001, 0.012]	0.003 [0.000, 0.009]	0.004 [-0.001, 0.013]	0.004 [-0.001, 0.012]	0.004 [0.000, 0.012]	0.003 [0.000, 0.008]	0.004 [-0.001, 0.013]	0.004 [-0.001, 0.012]
SVO -> SS -> NC -> MH	0.000 [-0.001, 0.001]	0.000 [0.000, 0.001]	0.000 [-0.001, 0.001]	0.000 [-0.001, 0.001]	0.000 [-0.001, 0.001]	0.000 [0.000, 0.001]	0.000 [-0.001, 0.001]	0.000 [0.000, 0.001]	0.000 [0.000, 0.001]	0.000 [0.000, 0.001]
SVO -> SS -> PC -> MH	0.005 [0.003, 0.010]	0.005 [0.002, 0.010]	0.005 [0.003, 0.010]	0.004 [0.001, 0.008]	0.005 [0.002, 0.009]	0.005 [0.002, 0.009]	0.005 [0.002, 0.009]	0.003 [0.001, 0.007]	0.005 [0.002, 0.010]	0.005 [0.002, 0.010]
Total association	0.288 [0.230, 0.346]	0.219 [0.165, 0.272]	0.252 [0.200, 0.307]	0.267 [0.214, 0.325]	0.277 [0.224, 0.336]	0.245 [0.191, 0.303]	0.295 [0.236, 0.349]	0.246 [0.190, 0.303]	0.273 [0.216, 0.334]	0.255 [0.202, 0.316]

Note: SVO = excessive short-video use; MH = mental-health outcome; SS = social support; PC = positive coping; NC = negative coping; BC = bias-corrected. Path models were estimated in the confirmation sample (n = 1,829) using standardized residuals and 1,000 bootstrap draws.

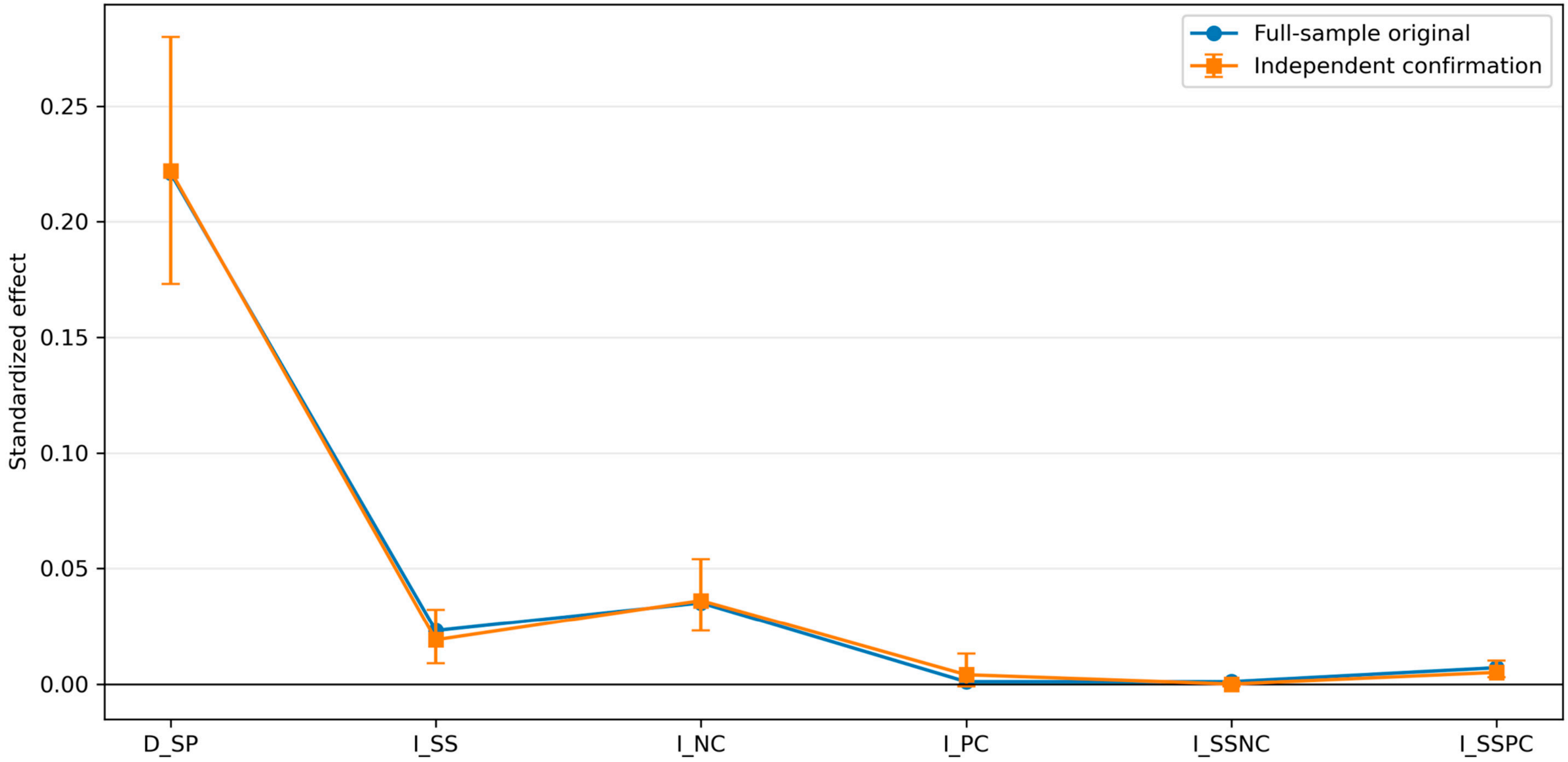

Figure S1. Comparison of direct and indirect associations in the full-sample and internal confirmation-sample total-score models.

Error bars represent bias-corrected bootstrap 95% confidence intervals. The confirmation analysis used a non-overlapping confirmation subset (n = 1,829) that did not contribute to model tuning or feature selection within the split-sample sensitivity analysis.
